# Anti-α-Internexin Autoantibody from Neuropsychiatric Lupus Induce Cognitive Damage via Inhibiting Axonal Elongation and Promote Neuron Apoptosis

**DOI:** 10.1371/journal.pone.0011124

**Published:** 2010-06-15

**Authors:** Xiao-ye Lu, Xiao-xiang Chen, Li-dong Huang, Chang-qing Zhu, Yue-ying Gu, Shuang Ye

**Affiliations:** 1 Department of Rheumatology, Shanghai JiaoTong University School of Medicine, Shanghai, China; 2 Department of Neurobiology, Shanghai Jiao Tong University School of Medicine, Shanghai, China; 3 Department of Emergency Medicine, Renji Hospital, Shanghai JiaoTong University School of Medicine, Shanghai, China; New York University, United States of America

## Abstract

**Background:**

Neuropsychiatric systemic lupus erythematosus (NPSLE) is a major complication for lupus patients, which often leads to cognitive disturbances and memory loss and contributes to a significant patient morbidity and mortality. The presence of anti-neuronal autoantibodies (aAbs) has been identified; as examples, anti-NMDA receptors and anti-Ribsomal P aAbs have been linked to certain pathophysiological features of NPSLE.

**Methods and Findings:**

In the current study, we used a proteomic approach to identify an intermediate neurofilament alpha-internexin (INA) as a pathogenetically relevant autoantigen in NPSLE. The significance of this finding was then validated in an expanded of a cohort of NPSLE patients (n = 67) and controls (n = 270) by demonstrating that high titers of anti-INA aAb was found in both the serum and cerebrospinal fluid (CSF) of ∼50% NPSLE. Subsequently, a murine model was developed by INA immunization that resulted in pronounced cognitive dysfunction that mimicked features of NPSLE. Histopathology in affected animals displayed cortical and hippocampal neuron apoptosis. In vitro studies further demonstrated that anti-INA Ab mediated neuronal damage via inhibiting axonal elongation and eventually driving the cells to apoptosis.

**Conclusions:**

Taken together, this study identified a novel anti-neurofilament aAb in NPSLE, and established a hitherto undescribed mechanism of aAb-mediated neuron damage that could have relevance to the pathophysiology of NPSLE.

## Introduction

Systemic lupus erythematosus (SLE) is the prototypic autoimmune disorder characterized by protean systemic manifestations and the presence of a wide spectrum of autoantibodies (aAbs). Currently, ∼200 aAbs have been identified in SLE and the number continues to grow [1]–[5]. However, only few of the aAbs have demonstrated clinical significance or value as biomarkers to facilitate diagnosis, disease activity assessment, disease phenotype dissection or prediction of prognosis. Furthermore, even fewer aAbs have established causal roles in the pathogenesis of SLE [6]–[9]. Therefore, most of the SLE-related aAbs were interpreted as the immune responses secondary to tissue injury and/or represent quantitative amplification of natural aAbs secondary to SLE polyclonal B cell activation [10]–[15].

Neuropsychiatric SLE (NPSLE) is a clinical feature of SLE attended by cognitive dysfunction and memory loss that contributes to significant patient morbidity and mortality [7], [16]–[19]. The presence of anti-neuronal aAb has been known in SLE for over 2 decades and several specific aAb potentially associated with NPSLE have been identified [2], [8], [20]–[30]. A seminal study was reported by Diamond and colleagues who demonstrated that a subset of anti-dsDNA from SLE patients binds NR2 glutamate receptors in the CNS [31], [32], and found that these aAb mediated cognitive impairment and emotional disturbances [33], [34]. Recently, another important finding demonstrated that anti-ribosomal P aAb could induce depression in mice via targeting a novel neuronal surface protein causing calcium influx and apoptosis [35], [36]. These findings support the hypothesis that certain aAbs against CNS autoantigens are pathogenic and display different mechanisms that could help understand the various NPSLE clinical phenotypes.

In the current study, we indentified the intermediate neurofilament alpha-internexin (INA) as a target antigen in NPSLE by using a proteomics approac\h. This finding was then validated in an expanded of a cohort of NPSLE patients and controls showing that significantly higher titers of aAb against INA are found in both the serum and more importantly, the cerebrospinal fluid (CSF) of NPSLE. Subsequently, a murine model was developed by INA immunization that bears pronounced cognitive dysfunction which mimics NPSLE. Brain tissue histopathology displayed cortical and hippocampal neuron apoptosis. In vitro studies further demonstrated that anti-INA Ab could mediate neuronal damage by inhibiting axonal elongation and driving the neurons to apoptosis. Taken together, this study identified a novel anti-neurofilament aAb in NPSLE, and established a hitherto undescribed mechanism of aAb-mediated neuron damage that could have relevance to the pathophysiology of NPSLE.

## Materials and Methods

### Ethics Statement

The study was approved by the Institutional Review Board of Renji Hospital. All subjects or their families gave written informed consent. All experimental protocols were approved by the Animal Care and Use Committee of Shanghai Jiao Tong University School of Medicine, the approval numbers for this study is 2007126 and 2008078. The cerebellar tissue slides of non-human primate were purchased from EUROIMMUN(BIOCHIP Mosaic™ Cerebellum, Germany).

### NPSLE Patients and Controls

Two hundred and fifty-six hospitalized patients admitted to the Departments of Rheumatology, Neurology, or Emergency Medicine, Shanghai Renji Hospital from January 5, 2005 to December 26, 2008, were enrolled in the study. All SLE were diagnosed according to the 1997 American College of Rheumatology (ACR) revised classification criteria. NPSLE (n = 67) was classified according to the ACR nomenclature and case definitions [16], [37], [38]. Control groups included (i) a separate group of SLE (n = 12) manifested as cerebral infarction (CI) with or without anti-phospholipid aAbs; (ii) SLE that had developed septic meningitis (n = 22) where the diagnosis was established when a specific bacterial or fungal pathogen was isolated in CSF; (iii) active SLE without CNS involvement (n = 85); (iv) rheumatoid arthritis (n = 30); (v) other neurological diseases (n = 40); and (vi) healthy donors (n = 81) where hospital staff and medical students served as normal controls. Serum samples from patients and healthy donors, and totally 177 CSF samples from NPSLE and disease controls were obtained. The characteristics of the study populations are summarized in Table 1. The majority of the CSF samples have been used in our published reports [39], [40].

**Table 1:** Characteristics of the study population.

Groups	Gender: M/F	Age: years (Mean±SD)	Sera/CSF: number	Descriptions
*NPSLE	8/59	29.5±11.6	67/60	Acute confusional state in 40 patients (22 complicated with seizure, 4 psychosis, 2 myelopathy, 2 seizure and psychosis, 2 seizure and myelopathy, 1 aseptic meningitis, 1 chorea, 1 myasthenia gravis), isolated cognitive dysfunction in 6 (2 with headache), seizure in 6, psychosis in 3, Demyelinating Syndrome in 2, movement disorder (chorea) and cranial neuropathy in 1, Guillain-Barré Syndrome in 1 and myelopathy in 7 patients.
SLE with Cerebral Infarction(CI)	3/9	35.5±12.1	12/10	6 with anti-phospholipid antibodies.
SLE Control	8/77	32.4±10.4	85/28	Active SLE without CNS manifestation.
SLE with Septic Meningitis	1/21	46.3±11.2	0/22	Mycobacterium tuberculosis in 13, Cryptococcus neoformans in 9 patients.
Rheumatoid Arthritis(RA)	4/26	36.7±13.3	30/0	**_**
Neuro-disease	25/15	42.9±14.5	0/40	cerebrovascular disease in 6, epilepsy in 2, migraine in 5, demyelinating disease in 4, spinal stenosis in 1, leukodystrophy in 1, myelopathy in 2, mild head trauma in 3, and CNS infection in 16 patients.
Normal Control	21/60	39.3±13.2	81/0	_

* All of the 67 NPSLE patients were evaluated by Glasgow or Mini Mental State Examination (MMSE) and showed conscious disturbance or a cognitive deficit.

### Animals

SD rats and C57BL/6 mice, all females, were purchased from Shanghai SLAC Laboratory Animal Co. Ltd. Pregnant SD rats were used for primary embryo neuron cell culture.

### Protein preparation, 2-D electrophoresis, immunoblotting and mass spectrometry

Brain and spinal cords were isolated from adult SD rats, and protein was extracted by a commercial protein-extraction kit (CALBIOCHEM Proteo Extract, 539779, Darmstadt, Germany). 2-DE was performed according to manufacturer's instructions (BIO-RAD, 163-2105, USA). After the electrophoresis, the gels were stained with Coomassie brilliant blue or used for protein transfer onto nitrocellulose membranes. Western blotting was performed as described previously [24], [26], [27], [41]. The serum samples were diluted to 1∶100 for immunoblotting the membranes and the bound antibodies were visualized by 5-Bromo-4-chloro-3-indolylphosphate/Nitroblue etrazolium (BCIP/NBT, CALBIOCHEM, 203790). The positive spot in the gel corresponding to the one identified on the nitrocellulose membranes were isolated, digested with trypsin and then subjected to MALDI-TOF MS (Applied Biosystems, USA) operated in the reflectron-delayed extraction mode (Central Research of Medical College of Fu Dan University, Shanghai). The peptide mass data were searched against the protein database SwissProt.

### Antigen cloning and antibodies

A commercial plasmid (pReceiver-Z0330-B01, Fulengen, China) containing an insert of the full length open reading frame of the human INA gene was transduced into E. coli BL21-DE3 (Novagen, Sweden), and the expressed recombinant protein was purified by Ni2+ resin (20 mM Tris buffer, pH 10.0) (File S1). Rabbit anti-human polyclonal antibodies to INA were generated by immunizing rabbits with recombinant INA (rINA), purified by protein A and affinity chromatography, and a control IgG was prepared in parallel (technical service provided by Chinese Academy of Science). Monoclonal antibodies to INA (Chemicon, MAB 5224, Temecula, CA), alkaline phosphatase-conjugated goat anti-human IgG (KPL, 05-10-06, Gaithersburg, MD, USA), anti-caspase 3 antibody (Santa Cruz Biotechnology, sc-22140, CA, USA, produced in Goat; and AbCam, ab13847, Cambridge, MA, USA, produced in Rabbit), FITC-conjugated goat anti-rabbit IgG (KPL, 02-15-06), TRITC-conjugated goat anti-mouse IgG (KPL, 03-18-09), TRITC-conjugated goat anti-Rabbit IgG (KPL, 03-15-06) FITC-conjugated donkey anti-rabbit IgG (Jackson ImmunoResearch, 711-095-152, West Grove, PA), TRITC-conjugated donkey anti-goat IgG (Jackson ImmunoResearch, 705-025-003), anti-His-tag polyclonal antibody (Cell Signaling, 2365, Danvers, MA), were used in experiments.

### Enzyme-linked immunosorbent assay (ELISA), immunoblotting and indirect immunofluorescence

Fifty µl of purified rINA (2 µg/ml) was coated onto 96-well micro-plates (Corning Costar, 9018, USA). After the plates were blocked with 1% gelatin in PBS, sera diluted at 1∶100, or undiluted CSF were then added. The anti-INA pAb diluted at 1∶10000 was used as the positive control. In the immunoblotting assay, rINA was subjected to 10% SDS-PAGE under reducing conditions and transferred onto nitrocellulose membranes. After blocking, the membrane was probed with serum (diluted from 1∶200 to 1∶1000) and CSF (dilution of 1∶ 10 or 1∶20). The ELISA and immunoblotting assays were performed according to standardized protocol. Mice, SD rats, monkey cerebral, spinal cord or cerebellar tissue slides (EUROIMMUN, BIOCHIP Mosaic™ Cerebellum, Germany), and SD rat embryo neuron substrate coverslips were used in indirect immunofluorescence study. Serum or CSF samples were diluted to 1∶100, or undiluted, respectively. The anti-INA pAb diluted to 1∶200 was used as the positive control. Secondary antibodies were conjugated to either FITC or TRITC as described above. In some experiments, serum was pre-incubated and absorbed with rINA [42], [43].

### Immunization Protocol

Six to 8 week old female C57BL/6 mice were immunized intraperitoneally (i.p.) with vehicle alone or 300 µg rINA, per mouse, per immunization, in 100 µl of saline. The first immunization was performed using Complete Freund's Adjuvant (CFA) (Sigma-Aldrich, F5581, St. Louis, USA). Four boosters were given at bi-weekly intervals in Incomplete Freund's Adjuvant (IFA) (Sigma-Aldrich, F5506). To facilitate the increased permeability of the blood-brain barrier, LPS (E. coli, 055:B5, Sigma), at a dose of 3 mg/kg diluted in lactated Ringers solution to 0.3 mg/ml, was given as an i.p. injection twice, 48 hr apart at 8 weeks after the last INA immunization. The histology, cognitive and behavioral studies were performed at 1–2 weeks after the LPS treatment. The experimental protocol was adapted from previous publications[31]–[33]. Animals were sacrificed after the cognitive test.

### Behavioral Assays

An experimental group (INA immunized, LPS-treated, n = 16) and two control groups (normal, n = 10 and INA immunized alone, n = 12) were studied. The studies included: the Morris water maze (spatial learning and reference memory), open-field activity, and beam-walking. The water maze task comprised two phases and lasted for 5 consecutive days [44], which included a place navigation test in the initial 4 days of testing and a probe trial on day 5. The data were collected as described previously [45].

### Immunohistochemical staining

Animals were sacrificed by deeply anesthetizing with sodium pentobarbital (i.p., 40 mg/kg of body weight) and then transcardially perfused with saline followed by a fixative containing 4% paraformaldehyde and 0.05% glutaraldehyde in 0.1 M phosphate buffer (pH 7.4). For paraffin sections, the brains were removed, post-fixed over 48 h in 4% paraformaldehyde, and then embedded in paraffin. Coronal brain sections of hippocampal and cortex tissue were cut at 5 um. The TUNEL assay for apoptosis was carried out by using ApopTag® Plus Peroxidase In Situ Apoptosis Detection Kit (Chemicon, S7101).

### Primary cultures of cortical neurons

Cultures of pure cortical neurons were obtained from E16-17 embryos of SD rats as previously reported [46], [47]. Single-cell suspensions were collected and plated into cell culture plates pre-coated with poly-L-lysine (Sigma-Aldrich, p2636) at a density of 0.5×10^5^ cells/well for a 96-well plate (for MTT reduction assay) or 0.3×10^5^ cells/well for a 24-well chamber slide (for immunocytochemisty), and cultured in Dulbecco's minimum essential medium (DMEM) containing 10% heat-inactivated fetal bovine serum and 5% heat-inactivated horse serum at 37°C in a humidified 5% CO_2_ incubator. After attachment, the medium was replaced by serum-free Neurobasal medium supplemented with 2% B27 and 2 mM glutamine (all purchased from Gibco, Grand Island, NY). Neuronal cultures were subjected to various concentrations of sterile anti-INA pAb or control IgG. The neurite length assessment and cell viability was performed 24 or 96 hours later. In some experiments, caspase-3 fmk Inhibitor Z-DEVD (R&D, FMK004) at final working concentrations of 100 uM was added to the cell culture.

### Immunocytochemistry

The MTT (3(4,5-dimethylthiazol-2-yl)2,5-diphenyltetrazolium bromide)(Sigma-Aldrich) assay was used to study the reduction capacity of cortical neurons cultured *in vitro* as described previously [46], [47]. Survival of control group neurons was defined as 100% and the treated groups were expressed as percent of control group in MTT assay. Other cells were fixed with 4% paraformaldehyde, blocked with 10% normal goat serum for 20 min, permeabilized with 0.2% Triton X-100 for 10 min, and then washed three times with 0.01 mM PBS. Monoclonal antibody against INA, anti-caspase 3 antibody, Hoechst 33342 to stain nuclei (Sigma-Aldrich, B 2261), and then secondary antibodies were applied in cell staining. Some cultures were stained with secondary antibody alone. The coverslips were rinsed and mounted with Gel/Mount aqueous anti-fade mounting media (Biomeda Corp). The results of immunostaining were examined with an Olympus BX 60 microscope or confocal laser scanning microscope (ZEISS LSM 510 META). Image-Pro Plus software was used to allow quantitative analysis. The longest neurite from each single cell of different groups was measured at 24 h after pAb treatment. 200 cells from each preparation were randomly selected and each experiment was performed in triplicate cover slips, more than 600 cells of each cell group per experiment were measured.

### Statistical analysis

Data were expressed as mean±S.E.M. Student's t-test, ANOVAs with repeated measures, and one-way ANOVA followed by post hoc Dunnett's t-test were used for statistical evaluation of differences between groups. An alpha level of 0.05 was used as the criterion for significance.

## Results

### Identification of the 56 kDa internexin as target autoantigen in NPSLE

**Figure 1:**
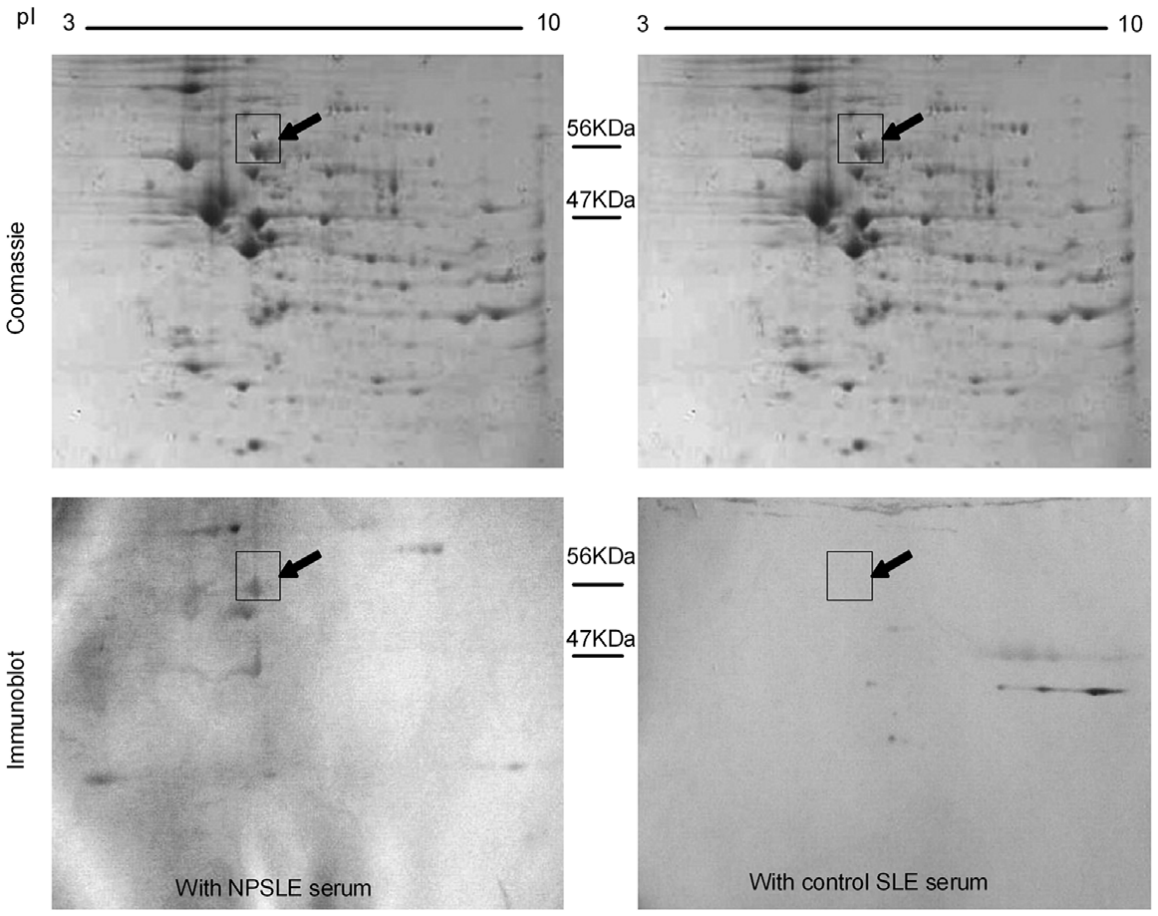
Identification of α-internexin (INA) as target antigen in NPSLE serum. The proteins extract from rat brain and spinal cord were separated by 2-DE and stained with colloidal Coomassie blue (upper panels), or immunoblotted with NPSLE (lower left panel) or control (lower right panel) sera. An immunoreactive spot at ∼56 kDa/*pI* 5.2 was excised and subjected to MALDI-TOF MS. By searching the protein database SwissProt, the peptide mass-mapping matched rat α-internexin (INA, alpha;α-INA; Rattus norvegicus, gi|9506811, kDa 56081.6, PI 5.2, protein score 99.99%). The data is representative of 4 independent experiments.

Our studies began by utilizing the brain or spinal cord tissue sections from mice, rat, and monkey as substrates for screening anti-neuronal aAbs in the sera of NPSLE patients by indirect immunofluorescence. Four NPSLE sera (two patients with severe cerebralitis and myelitis, one with seizures, one with psychosis) were identified by their reactivity to all species (Figure S1). All sera probes are ANA positive without cytoplasmic staining against Hep2 cells, and neither have anti-ribsomal P antibodies. Brain and spinal cord proteins of the Sprague-Dawley (SD) rat were extracted, then resolved by 2-dimention electrophoresis (2-DE) and immunoblotted with these four sera, all of which recognized a protein spot at 56 kDa/pI 5.2 (Figure 1). The spot was excised from the gel, cleaved with trypsin, and subjected to matrix-assisted laser desorption ionization time of flight mass spectrometry (MALDI-TOF MS). The determined peptide mass data with the highest score matched rat α-internexin (INA) (SwissProt accession no. Theoretical *M*r  = 56115.6 and p*I*  = 5.20). BLAST analysis revealed that INA is highly conserved from rodents to Homo sapiens (91–94% homology) [48]–[50]. Of note, it has been reported that there is no cross antigenicity of INA with other neurofilament proteins [51], [52].

### Anti-INA is a novel aAb in the sera and CSF of NPSLE and the CSF anti-INA levels correlate with cognitive impairment

**Figure 2:**
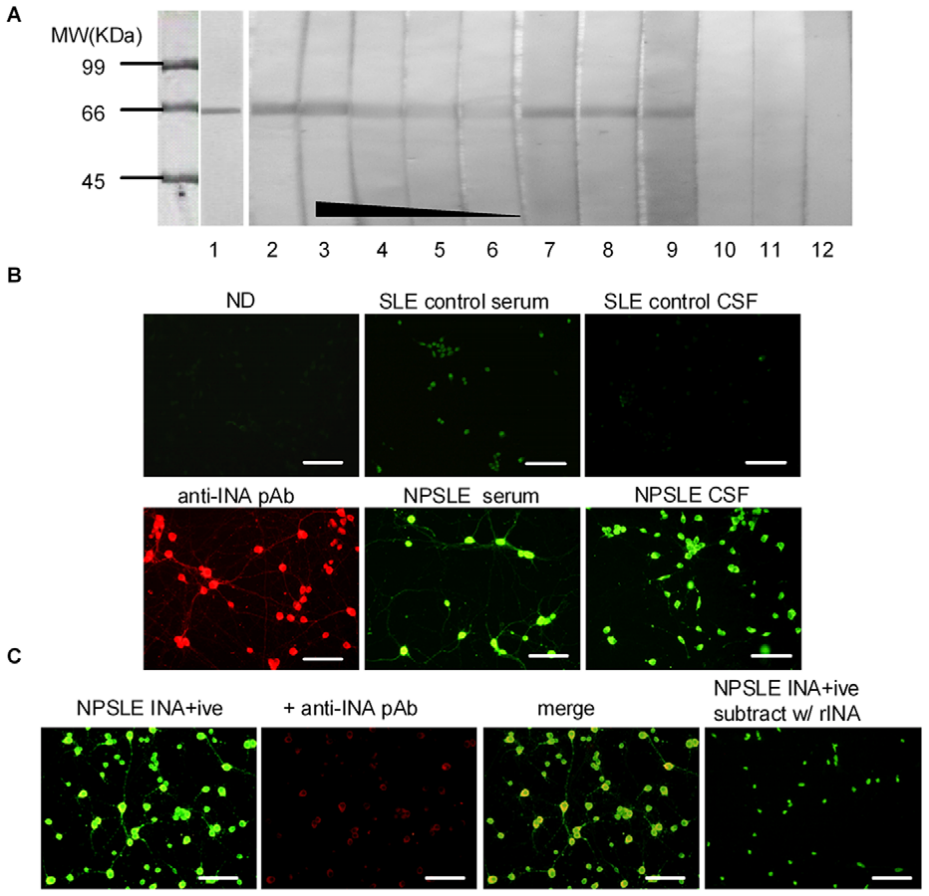
Anti-INA aAb in the sera and CSF of NPSLE. (A) Purified His-tagged recombinant human INA (66 kDa) was blotted with anti-His antibody (lane 1), anti-INA pAb (lane 2), NPSLE serum probe at serial dilutions of 1∶200, 1∶400, 1∶800, 1∶1000 (lane 3–6), the same patient's CSF diluted in 1∶10 and 1∶20 (lane 7–8), another NPSLE serum probe and the patient's CSF (lane 9–10), CSF from a SLE control and a neurological disease control (lane 11–12). (B) SD rat primary neuron substrate stained with serum of normal donor (ND), anti-INA pAb, serum or CSF from SLE patients as indicated. (C) NPSLE serum probe competitive inhibit anti-INA pAb staining. After pre-incubated with recombinant INA (rINA), the serum probe lost its cytoplasmic staining.

To confirm that anti-INA aAbs are found in the sera of NPSLE patients, purified His-tagged recombinant human INA was used in a western blot. As expected, sera probes have a dose-dependent reactivity to INA as opposed to control samples (Figure 2A). Of note, anti-INA aAb was also detected in 3 of 4 CSF from the probe NPSLE. In a parallel indirect immunofluorescence assay, anti-INA positive serum/CSF probes from NPSLE reacted with primary cultured rat cortical neurons, with a distinctive cytoplasmic and neurite staining pattern compared to a faint anti-nuclear staining pattern from control SLE samples (Figure 2B). Anti-INA polyclonal antibody (pAb) obtained from rabbit showed a similar staining pattern as the probes, and when double-stained with the probe, a co-localization and competitive effect can be observed under fluorescent microscope. Finally, pre-incubated with INA, the probes lost their cytoplasmic staining to neurons (Figure 2C).

**Figure 3:**
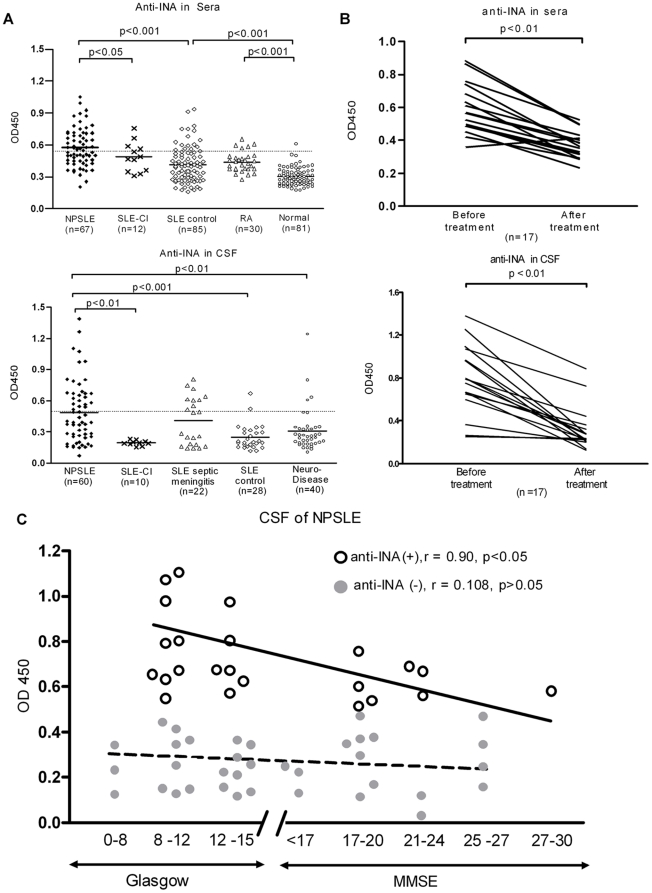
Anti-INA aAb in the sera and CSF is correlated with NPSLE activity. (A) Anti-INA aAbs in sera or CSF were determined by ELISA. SLE-CI  =  SLE with cerebral infarction. (B) Dynamic changes anti-INA Ab levels in the sera or CSF from NPSLE patients before and after induction therapy which lead to clinical remission. (C) The NPSLE patients who have positive anti-INA Abs in CSF, the titer of the Ab was inversely correlated with the cognitive status as measured by Glasgow coma scale or MMSE.

After the thorough confirmation of the existence of anti-INA aAb in NPSLE, a further validation ELISA was carried out in a cohort of patients (Figure 3A). We tested for anti-INA aAbs in the sera of NPSLE (n = 67), SLE manifested as cerebral infarction (CI) with or without aPL aAbs (n = 12), active SLE without CNS involvement (n = 85), rheumatoid arthritis (RA) patients (n = 30) and normal controls (n = 81), and the positive anti-INA Ab rates in sera among aforementioned groups were 52.2% (35/67), 33.3% (4/12), 18.8% (16/85), 13.3% (5/30) and 1.2% (1/81), respectively. The different frequencies of anti-INA Ab between NPSLE group and other control groups was statistically significant (*p*<0.001). More importantly, the OD of anti-INA Ab in CSF of NPSLE patients was significant higher than that of SLE-CI, SLE controls and neurological diseases controls. At a cutoff value determined by the average OD of SLE controls plus 2SD, the rates of positive anti-INA in CSF of NPSLE (n = 60), SLE-CI (n = 12), SLE controls (n = 28), neurological disease controls (n = 40) were 41.7% (25/60), 0% (0/12), 7.1% (2/28), 10% (4/40), respectively. Unexpectedly, 40.9% (9/22) SLE with CNS infections also had detectable anti-INA Ab in CSF. Nevertheless, longitudinal analysis of both sera and CSF among 17 NPSLE patients showed that the levels of anti-INA Ab significantly declined after initiation of therapy and eventual clinical remission (Figure 3B). The titer of the Ab was inversely correlated with the cognitive status as measured by Glasgow coma scale or Mini Mental State Examination (MMSE) in the NPSLE patients who had anti-INA Abs in their CSF (Figure 3C).

### Mice immunized with INA plus LPS display cognitive impairments that mimic NPSLE

**Figure 4:**
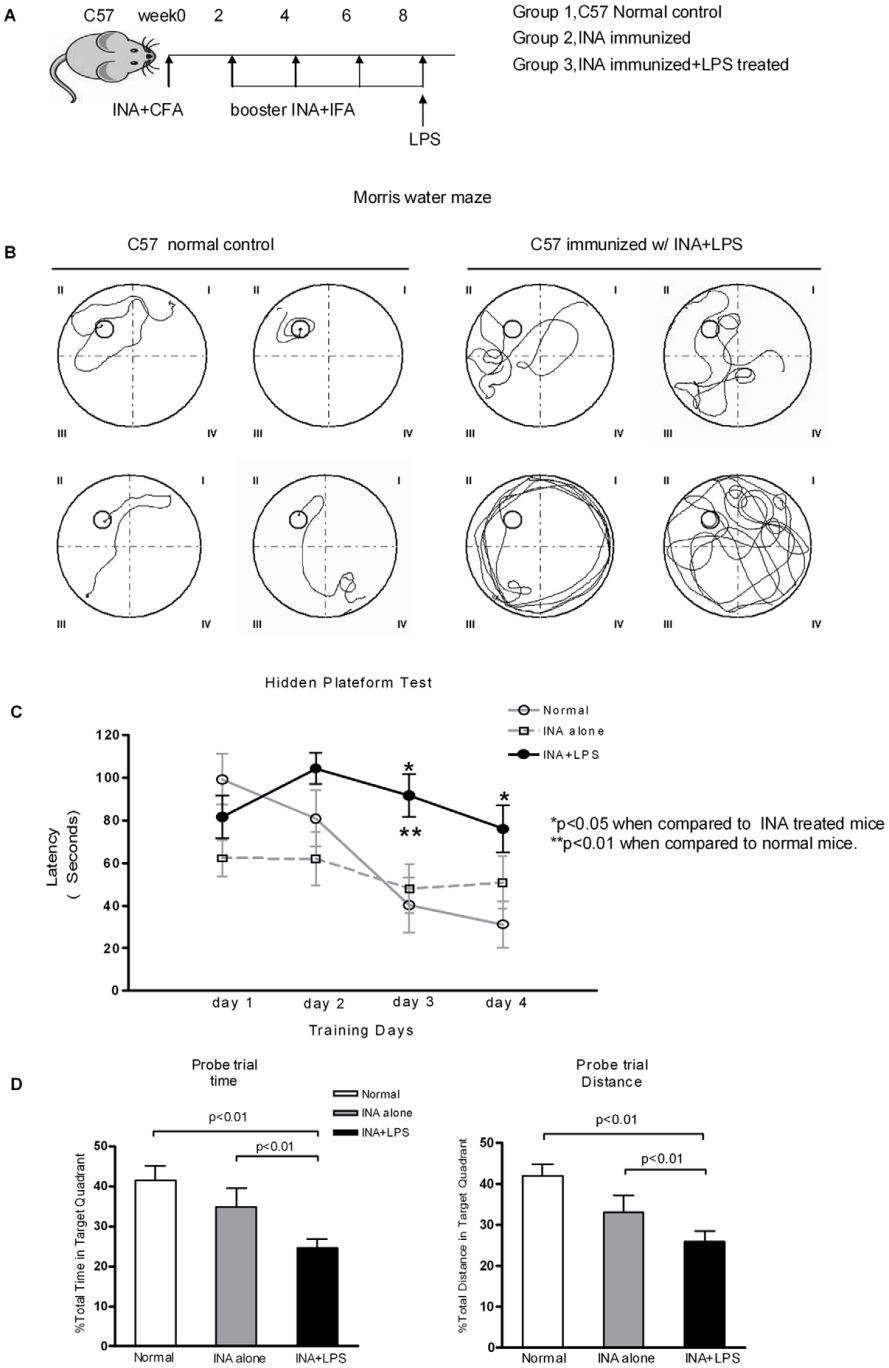
Mice immunized with INA plus LPS display cognitive impairments. (A) Immunization protocol in C57 mice. Normal control (n = 10), INA immunized (n = 12), INA+LPS treated (n = 16). (B) Morris water maze test. Representative swimming traces of mice from different groups on the third training day. The hidden platform is located in quadrant II. (C) The time latency to find the hidden platform in different groups of mice during consecutive 4 training days. (D) The platform was removed at day 5, and the time (left) and efforts (right) mice spent in the quadrant where the previous platform placed were calculated. All data were presented as mean±S.E.M.

A key question was whether anti-INA Abs are pathogenic or merely a coincidental phenomenon. To address this question, a murine model was established in order to assess the *in vivo* effects of anti-INA on CNS (Figure 4A). Briefly, all mice immunized with INA developed high titer anti-INA Abs 8–9 weeks after immunization (data not shown). Only the mice immunized with INA plus LPS, which facilitates increasing the permeability of the blood-brain barrier (BBB), displayed distinctive learning and memory deficits on tasks that depend on the integrity of the hippocampus and cortex as gauged by Morris water maze test (Figure 4B). The time latency for the mice finding a submerged hidden platform in the water maze remained prolonged over the 4 days of training in the INA+LPS group (Figure 4C). In addition, when the platform was removed at day 5, the control groups of mice spent more time and effort in the quadrant where the previous platform placed, as opposed to the INA+LPS group thus suggesting a significant memory impairment (Figure 4D). This impairment in learning and memory function is consistent with the neuropsychiatric studies of lupus-prone murine models which demonstrate poor performance in spatial memory tasks [53]–[57]. Other profound neurological dysfunction, such as exploratory behavior and motor coordination, was also observed in INA+LPS mice in open field activity and beam walking tasks (Figure S2).

### INA immunized and LPS treated mice exhibited hippocampal and cortical neuron apoptosis

**Figure 5:**
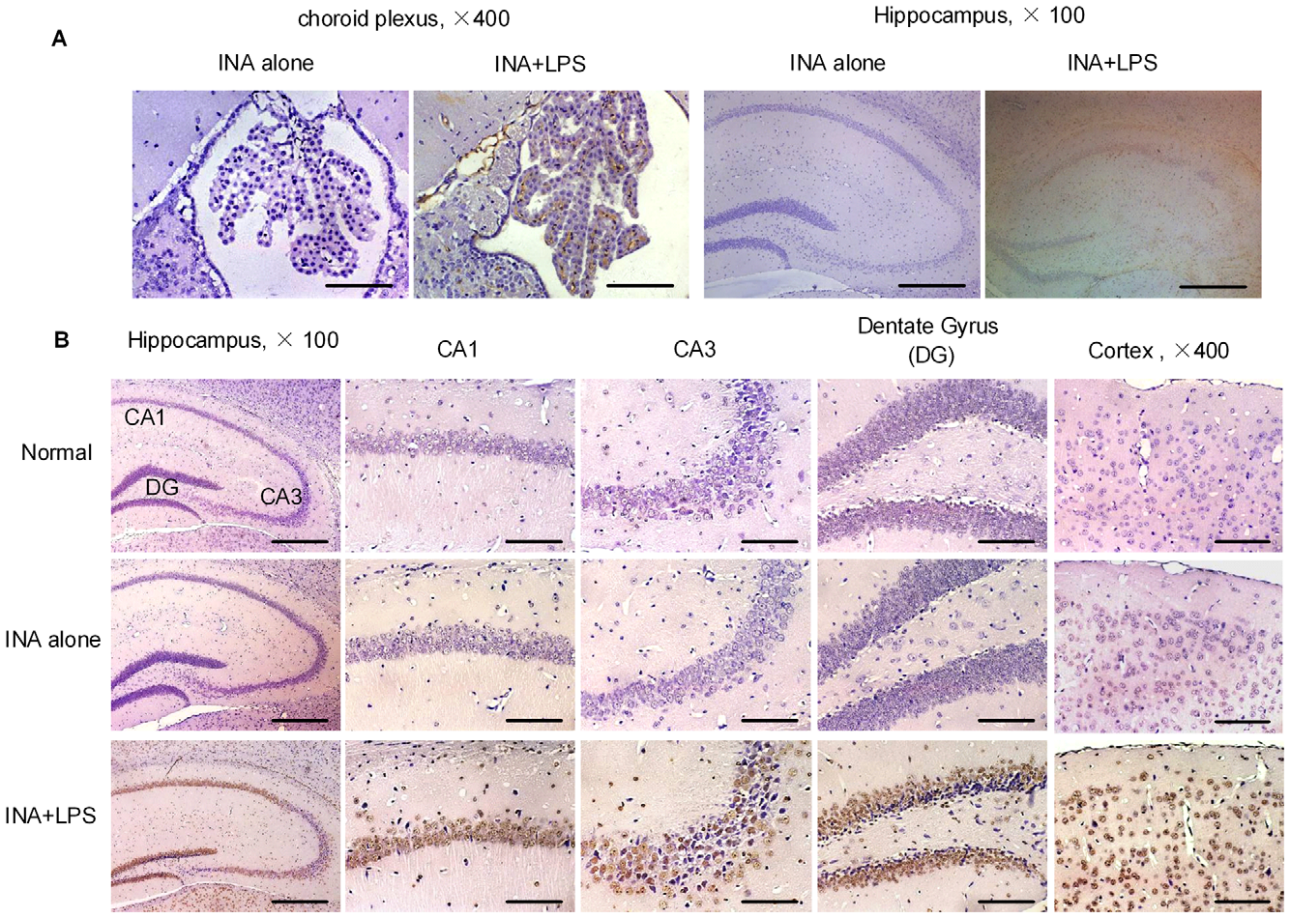
INA immunized plus LPS treated mice exhibited hippocampus and cortex neuron apoptosis. (A) IgG deposition at the choroid plexus (left) and hippocampus (right) in different groups of mice. (B) CA1, CA3 and dentate gyrus regions of hippocampal, and cortex areas from indicated group of mice. TUNEL staining demonstrated neuron apoptosis. (×400, Bar  = 50 µm; ×100, Bar  = 200 µm).

As evidence that anti-INA Ab indeed penetrated the BBB and gained access to its cognate CNS targets, it was observed that there was IgG staining of the choroid plexus in INA+LPS treated mice (Figure 5A). The effects of anti-INA penetration can be appreciated as prominent diffuse hippocampal (CA1, CA3 and dentate gyrus regions) and cortical neuron apoptosis as gauged by TUNEL staining in INA+LPS treated mice, but not in mice immunized with INA alone or in sham controls (Figure 5B).

### The anti-INA pAb inhibit the growth of neurites and result in neuron apoptosis in vitro

**Figure 6:**
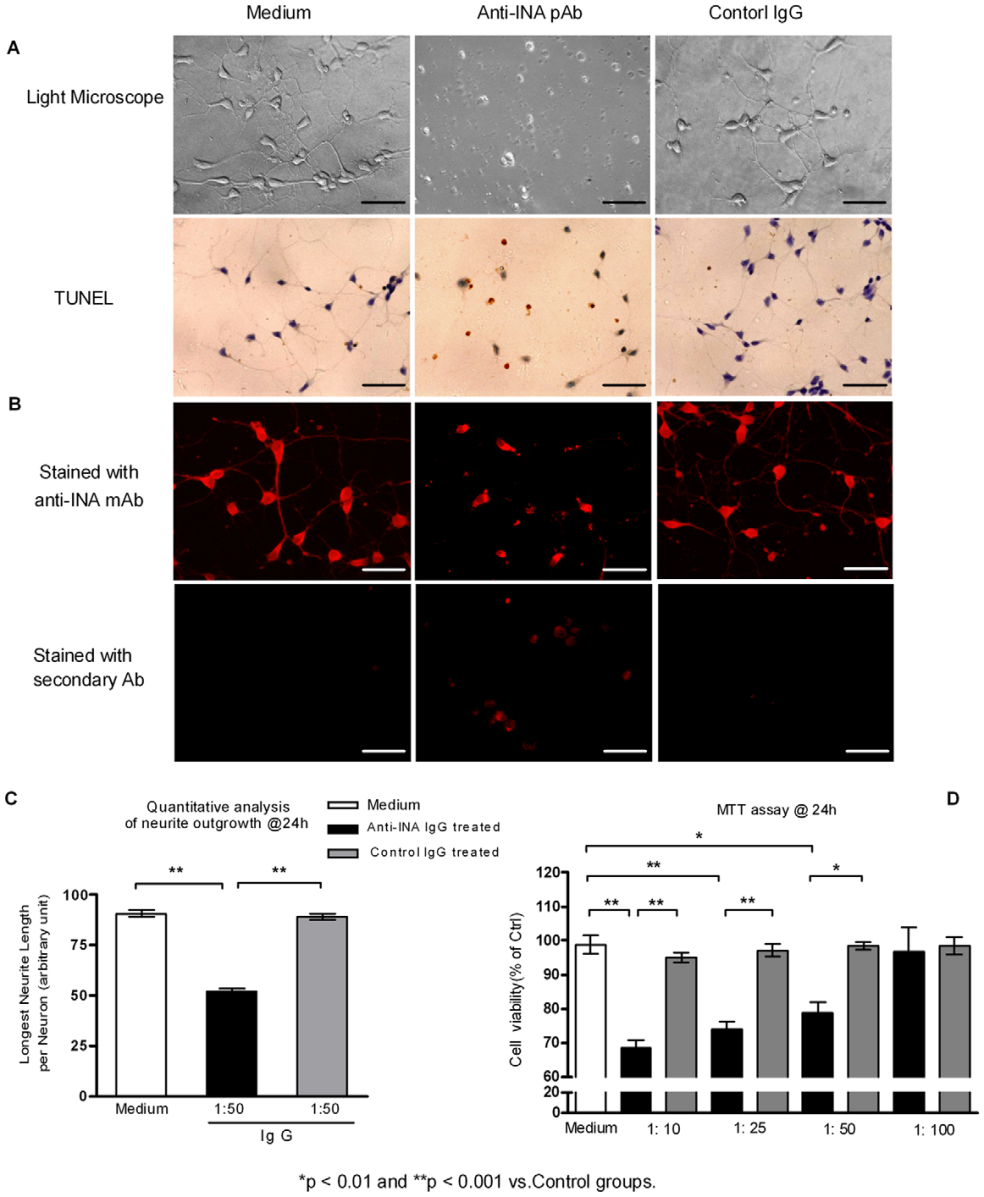
The anti-INA pAb inhibit the growth of neurites and result in neuron apoptosis in vitro. (A) Primary rat embryo neurons were co-cultured with medium, sterile anti-INA pAb (1∶50), or control IgG for 96 hrs. The neurite outgrowth and cell viability was observed by light microscopy. Apoptotic cells were detected by TUNEL assay. (B) A monoclonal antibody (Mab 5224) against INA was used for displaying INA location. The TRITC goat anti-rabbit secondary antibody staining was to demonstrate the internalization of co-cultured Abs during the neuron cell growth. (Bar  = 25 µm) (C) Quantitative measurement of cultured neurons axonal length at 24 hrs. (D) Viability of primary cultured neurons subject to indicated concentrations of anti-INA pAb or control IgG after 24 h. The percentage of cell survival normalized to control cells was applied in MTT assay. Data are represented as mean±SEM of three independent experiments.

To determine whether there is a pathogenic effect of anti-INA Ab on neurons, rat embryo cortical neurons were subjected to anti-INA pAb or control IgG. After 96 h of culture, cells exposed to anti-INA pAb displayed a significant neurotoxicity as gauged by impeded axonal outgrowth and cell viability (TUNEL staining) (Figure 6A). Of note, anti-INA pAb, but not control IgG, could penetrate the cells as demonstrated by directly secondary antibody staining. As an intermediate neurofilament component, the normal staining pattern of INA by an anti-INA monoclonal Ab (mAb) was characterized within the cytoplasm of the cells and also in the cell processes (Figure 6B, also seen in Figure 2B–C). Seemingly related to these observations, it was noted that cells treated with anti-INA pAb sequestrated INA in a cytoplasmic compartment surrounding the nuclei.

**Figure 7:**
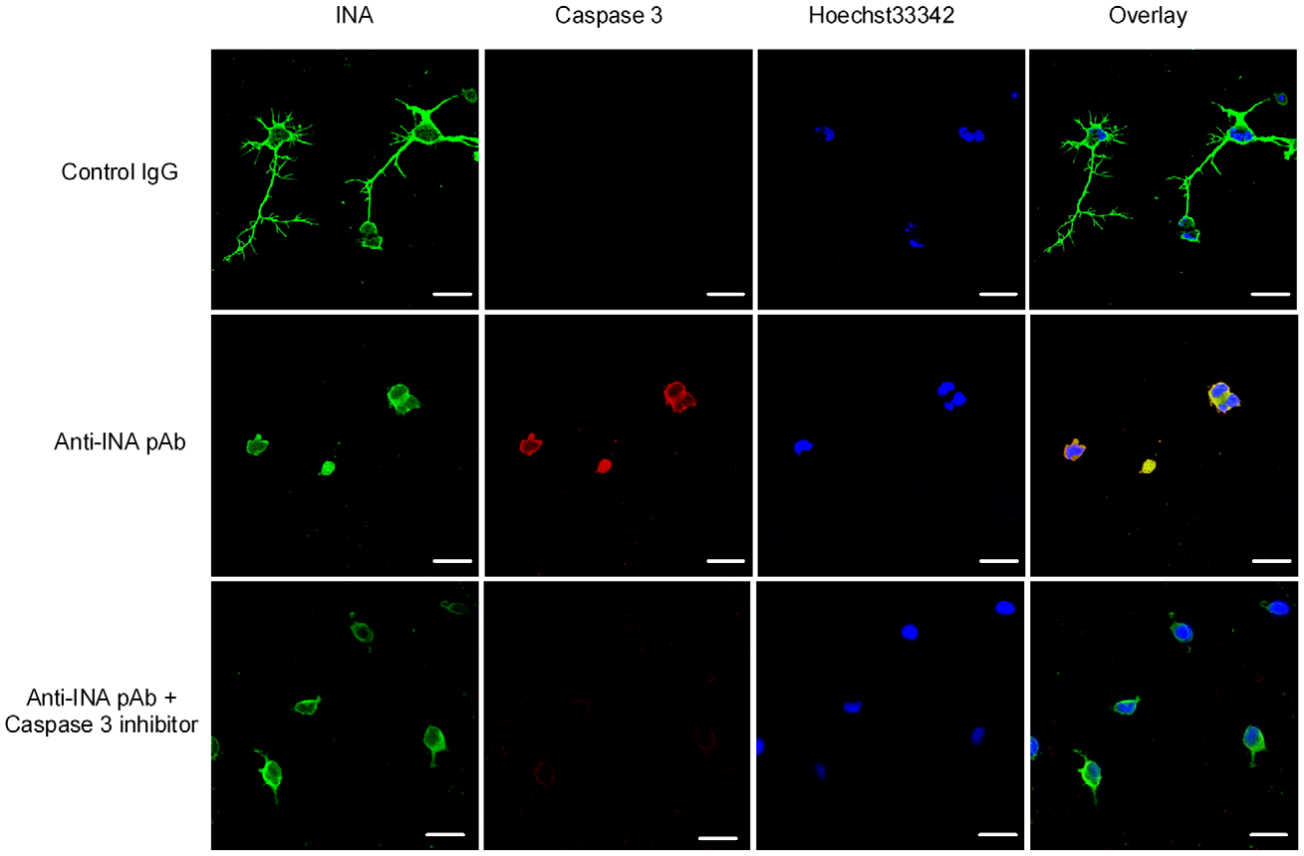
Apoptosis with caspase-3 activation is the downstream effect of anti-INA mediated neuron damage. Primary rat cortical neurons were co-cultured with anti-INA pAb or control IgG for 24 h. Caspase-3 inhibitor was added as indicated. The cells were triple-labeled Hoechst (blue), caspase-3 (red), and INA (green). (Scale bar  = 10 µm).

The neurotoxic effect of anti-INA pAb could be appreciated as early as the 24^th^ hour of culture. Quantitative measurement of neurite length illustrated a significant reduction by ∼50% after 24 h exposure of anti-INA pAb (Figure 6C). In parallel, the MTT assay showed that anti-INA pAb impaired neuron viability in a dose-dependent manner (Figure 6D). Furthermore, the anti-INA pAb-induced neuron apoptosis process was detected by Caspase-3 activation. The perfectly overlapped co-localization of Caspase-3 and INA in the cytoplasm indicated a possible link between those two molecules. Interestingly, when Caspase-3 was blocked by a specific inhibitor, the neurite outgrowth was still impeded by anti-INA pAb, which suggested that apoptosis is the downstream effect of anti-INA mediated neuron damage (Figure 7).

## Discussion

In the current study, alpha-internexin was identified as a new target autoantigen for NPSLE, in that the anti-INA aAbs in the sera of NPSLE patients are significantly increased in ∼50% of NPSLE sera. More importantly, even by using SLE control values (mean±2SD) as cut-off, anti-INA Ab is still positive in >40% of the CSF of NPSLE, which indicated a strong association between this aAb and an intrathecal autoimmune processes. In addition, the titer of anti-INA, both in the serum and CSF was correlated with disease activity, further suggesting its potential usefulness as a biomarker in NPSLE. However, its specificity and clinical value may have limitations as evidenced by a similar positive rate in the CSF of SLE patients with CNS infections [58]–[60]. It is noteworthy that the pathogenesis of NPSLE is likely multi-factorial and that antibodies to other targets may work independently or in concert to produce the various NPSLE clinical phenotypes.

INA is a constitutional intermediate neurofilament (IF) protein first described in 1985 [61].

In the mammalian nervous system, three types of IF proteins are expressed in differentiated neurons, which include light (NF-L), medium (NF-M), heavy (NF-H) neurofilament triplet proteins (NFTPs). INA is expressed abundantly to form the group of type IV IF throughout the development of the peripheral and central nervous system, and is restricted to the central nervous system in the adulthood [62]–[65]. Neuro-cytoskeletal filament proteins ingeneral [59], [60], or their components such as glial fibrillary acid protein [3], microtubule associated protein-2 [27], [66], and more recently, INA have been described as potential target autoantigens in SLE[58]. However, due to lack of sufficient validation and CSF aAb analyses in the aforementioned studies, aAbs against neurofilament proteins in NPSLE have been considered somewhat insignificant [67].

Our data not only demonstrated a link between anti-INA aAbs and NPSLE, but also took one step further to illustrate a direct neurotoxic effect of this aAb. A murine model displayed anti-INA access to its target protein when the permeability of the BBB was interrupted by LPS, resulting in profound cortical and hippocampal dependent cognitive impairment/memory loss. A plausible mechanistic explanation of the pathological NPSLE phenotype was supported by the observations that anti-INA Ab could inhibit neurite outgrowth and axonal elongation, and subsequently induced neuron apoptosis both *in vivo* and *ex vivo*.

It is known that, in adult CNS, INA is selectively expressed in the gray and underlying white matter, such as neuron cell bodies and processes of cortical layer II neuron, CA1 subfield and granule neurons of the dentate gyrus of hippocampus, cerebellar granule cells, and thin-caliber parallel fibers, as well as in the basal ganglia, thalamus, midbrain, pons and gray matter of the spinal cord [52], [61], [65], [68]–[72]. The functions of INA include a scaffold for IF formation and regulating the expression of other neurofilaments during neuronal development, and it can self-assemble into homopolymers or coassemble with other IF proteins to form NFTPs in mature neurons [64], [65], [68], [69], [73]–[76]. The contribution of INA to cytoskeletal integrity is important for axonal calibre and nerve conduction speed, and INA plays an important role in facilitating the neurite outgrowth [64], [68], [77]–[79]. Of potential relevance to our findings, it is well established that abnormal accumulation or mislocation of neurofilaments is a pathological hallmark of many human neurodegenerative disorders including amyotrophic lateral sclerosis, dementia with Lewy bodies, and Parkinson disease.More specifically, INA along with other NF proteins, are found to form pathological inclusions in a newly defined neuronal intermediate filament inclusion disease (NIFID), which presented as progressive early-onset dementia, pyramidal, and extrapyramidal dysfunctions [72], [80]. Studies with transgenic mice overexpressing IF proteins, including INA, provide evidence that dis-organization of the IF network can cause neurofilament misaccumulation, progressive neurodegeneration, and ultimate loss of neurons [73]. The findings that genetically modified mice for IF successfully mimic certain degenerative neuropathological phenotype [81]. It was also found that overexpression of INA in PC12 cells induced apoptosis-like cell death [78]. Taken together, these data support our observations that anti-INA Ab may interfere with the location and function of INA, which in turn contribute to neuronal damage.

Nevertheless, the precise mechanism of anti-INA mediated neuron damage remains unclear. Previous studies suggested that aAb induced neuronal death can occur without complement activation or Fc receptor engagement, and demonstrated that aAbs can alter behavior without causing inflammation in the CNS [32]–[35]. The role of aAb directed against NFTPs or their individual subunits in certain neurodegenerative conditions has also been implicated [82]. Although according to previous literatures that autoantibodies have been shown to penetrate various living cells, how anti-INA internalize into neurons and gain access to target proteins is still not known [83]–[86]. The possibilities of that it is LPS alters the localization of INA or allows anti-INA into cells can not be excluded, in addition, there may be another cross reactivity exists which plays a role in the anti-INA internalization process. Another key question needs to be answered is that how anti-INA Ab inhibits neurite outgrowth and drives the neuron to programmed death. A plausible explanation would be a two step process according to our data. First, anti-INA binds to INA, therefore interfere INA assembly/coassembly for neurofilament transportation, which result in INA accumulation and failure of axonal elongation. Subsequently, caspase 3 dependent apoptosis is activated due to neuron cytoskeleton “strangulation” [87], [88]. Further investigations are required to address these questions.

## Supporting Information

File S1 Gene information, ORF sequence information, and vector information of EX-Z0330-B01.
(0.06 MB DOC)

Figure S1 Screening anti-neuronal aAbs in the sera/CSF of NPSLE and SLE control patients by indirect immunofluorescence. NPSLE serum from a patients with severe cerebralitis and myelitis was identified by its reactivity to all species. All sera/CSF of SLE probes are ANA positive without cytoplasmic staining against Hep2 cells, and neither have anti-ribsomal P antibodies. Scale bar  = 50 µm.
(2.09 MB TIF)

Figure S2 An experimental group (INA immunized, LPS-treated, n = 16) and two control groups (normal, n = 10 and INA immunized alone, n = 12) were studied. Neurological dysfunctions including exploratory behavior and motor coordination, was observed in INA+LPS mice in open field activity and beam walking tasks.
(0.20 MB TIF)
