# Supplementary material for: Anti-α-Internexin Autoantibody from Neuropsychiatric Lupus Induce Cognitive Damage via Inhibiting Axonal Elongation and Promote Neuron Apoptosis
Source: PLoS One. 2010 Jun 15;5(6):e11124. doi: 10.1371/journal.pone.0011124 (PMC2886066; doi:10.1371/journal.pone.0011124)

**OmicsLink™ Expression Clone Datasheet of EX-Z0330-B01**

**Gene Information**

| Catalog No.: EX-Z0330-B01 |
| --- |
| Accession No.: BC006359     ORF Length:  1500BP |
| Vector size:  5548 BP (backbone only, ORF insert not counted) |
| Description: Homo sapiens, clone MGC:12702 IMAGE:4125949, mRNA, complete cds. |
| Vector: pReceiver-B01      Antibiotic for plasmid reproduction in E. coli: Ampicillin |
| Suggested Sequencing Primers:  Forward: 5'-TAATACGACTCACTATAGGG-3' Reverse: 5'-TTCACTTCTGAGTTCGGCATG-3' |

**ORF Sequence Informatioin of EX-Z0330-B01**

>EX-Z0330-B01 ORF sequence

ATGAGCTTCGGCTCGGAGCACTACCTGTGCTCCTCCTCCTCCTACCGCAAGGTGTTCGGGGATGGCTCTCGCCTGTCCGCCCGCCTCTCTGGGGCCGGCGGCGCGGGCGGCTTCCGCTCGCAGTCGCTGTCCCGCAGCAATGTGGCCTCCTCGGCCGCCTGCTCCTCGGCCTCGTCGCTCGGCCTCGGCCTGGCCTATCGCCGGCCGCCGGCGTCCGACGGGCTGGACCTGAGCCAGGCGGCGGCGCGCACCAACGAGTACAAGATCATCCGCACCAACGAGGAGGAGCAGCTGCAGGGCCTCAACGACCGCTTCGCCGTGTTCATCGAGAAGGTGCATCAGCTGGAGACGCAGAACCGCGCGTTGGAGGCCGAGCTGGCCGCGCTGCGACAGCGCCACGCTGAGCCGTCGCGCGTCGGCGAGCTCTTCCAGCGCGAGCTGCGCGACCTGCGCGCGCAGCTGGAGGAGGCCAGTTCGGCTCGCTCGCAGGCCCTGCTGGAGCGCGACGGGCTGGCGGAGGAGGTGCAGCGGCTGCGGGCGCGCTGCGAGGAGGAGAGCCGCGGACGCGAAGGCGCCGAGCGCGCCCTGAAGGCGCAGCAGCGCGACGTGGACGGCGCCACGCTGGCCCGCCTGGACCTGGAGAAGAAGGTGGAGTCGCTGCTGGACGAGCTGGCCTTCGTACGCCAGGTGCACGACGAGGAGGTAGCCGAGCTGCTGGCCACGCTGCAGGCGTCGTCGCAGGCCGCGGCCGAGGTGGACGTGACTGTGGCTAAACCAGACCTGACCTCGGCTCTGAGGGAGATCCGCGCCCAGTATGAGTCCCTGGCCGCTAAGAACCTGCAGTCCGCGGAAGAATGGTACAAGTCCAAGTTTGCCAACCTGAACGAGCAGGCGGCGCGCAGCACCGAGGCCATCCGGGCCAGCCGCGAGGAGATCCACGAGTATCGGCGCCAGCTGCAGGCGCGCACCATCGAGATCGAGGGCCTGCGCGGGGCCAACGAGTCCTTGGAGAGGCAGATCCTGGAGCTGGAGGAGCGGCACAGTGCCGAGGTAGCTGGCTACCAGGATAGCATTGGGCAGCTGGAGAATGATCTGAGGAACACCAAGAGTGAGATGGCACGCCACCTTCGGGAATACCAGGACTTGCTCAATGTCAAAATGGCTCTTGACATTGAGATAGCAGCTTACAGGAAACTGCTGGAAGGCGAGGAGACACGTTTTAGCACCAGTGGGTTAAGCATTTCGGGGCTGAATCCACTTCCCAATCCAAGTTACCTGCTCCCACCTAGAATCCTCAGTGCTACAACCTCCAAAGTCTCATCCACTGGGCTATCACTTAAGAAAGAGGAGGAGGAGGAGGAGGCATCTAAGGTAGCCTCTAAGAAAACCTCCCAGATAGGGGAAAGTTTTGAAGAAGTATTAGAGGAGACAGTAATATCTACTAAGAAAACCGAGAAATCAAATATAGAAGAAACCACCATTTCAAGCCAAAAAATATAG

Vector Information of EX-Z0330-B01


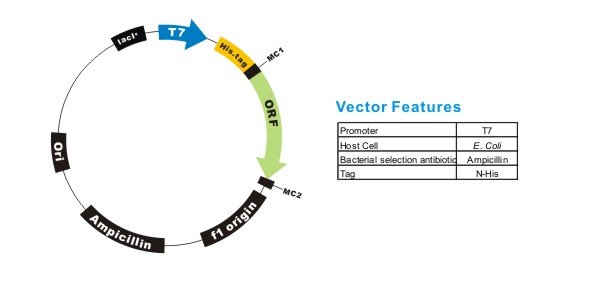


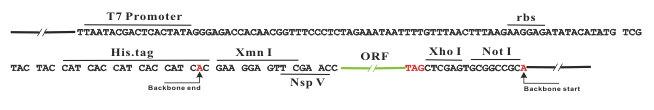

Supplement: File S1 — Gene information, ORF sequence information, and vector information of EX-Z0330-B01. (0.06 MB DOC) [file pone.0011124.s001.doc]
