# Supplementary figures and images for: Anti-α-Internexin Autoantibody from Neuropsychiatric Lupus Induce Cognitive Damage via Inhibiting Axonal Elongation and Promote Neuron Apoptosis
Source: PLoS One. 2010 Jun 15;5(6):e11124. doi: 10.1371/journal.pone.0011124 (PMC2886066; doi:10.1371/journal.pone.0011124)

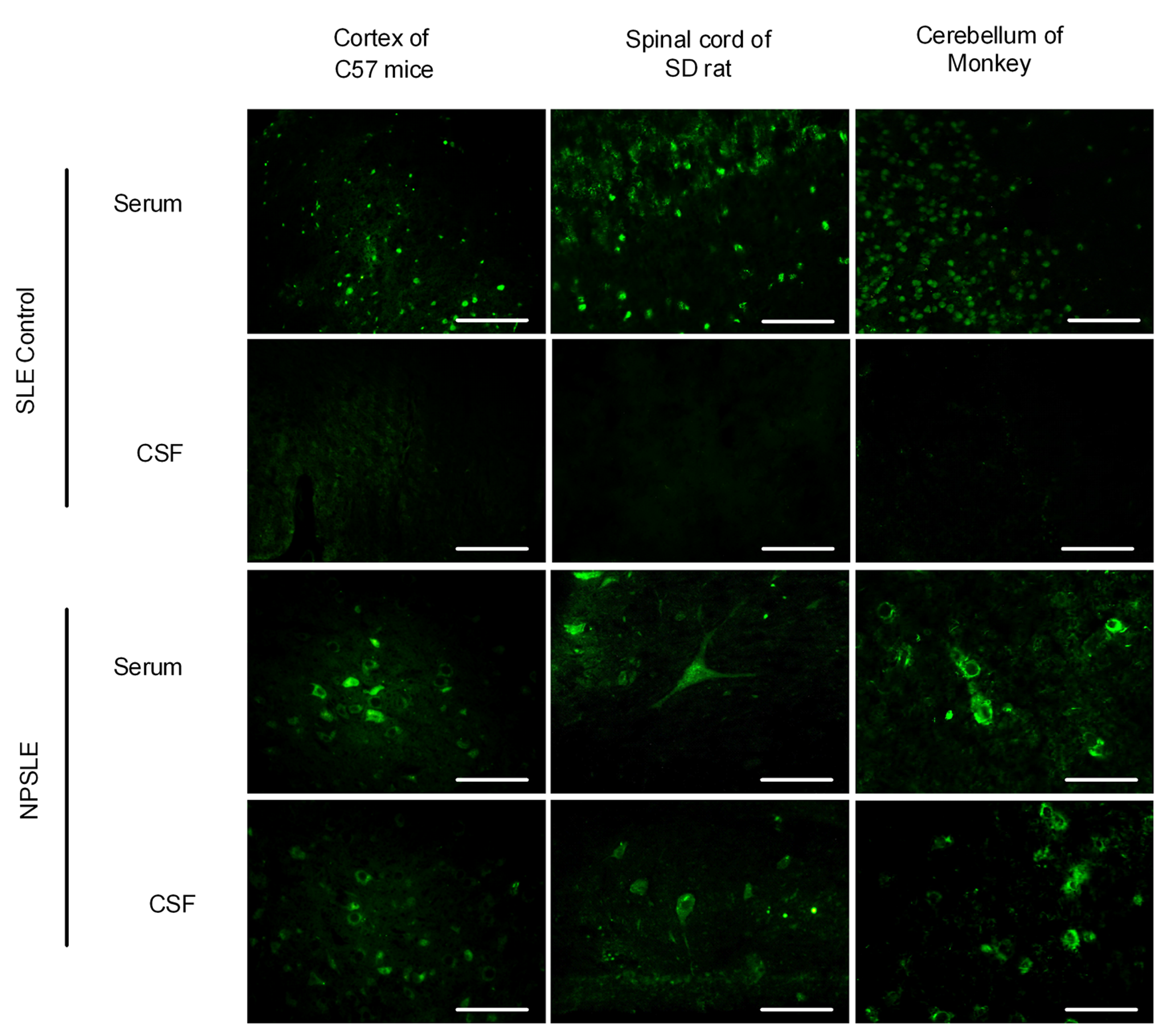

Supplement: Figure S1 — Screening anti-neuronal aAbs in the sera/CSF of NPSLE and SLE control patients by indirect immunofluorescence. NPSLE serum from a patients with severe cerebralitis and myelitis was identified by its reactivity to all species. All sera/CSF of SLE probes are ANA positive without cytoplasmic staining against Hep2 cells, and neither have anti-ribsomal P antibodies. Scale bar = 50 µm. (2.09 MB TIF) [file pone.0011124.s002.tif]

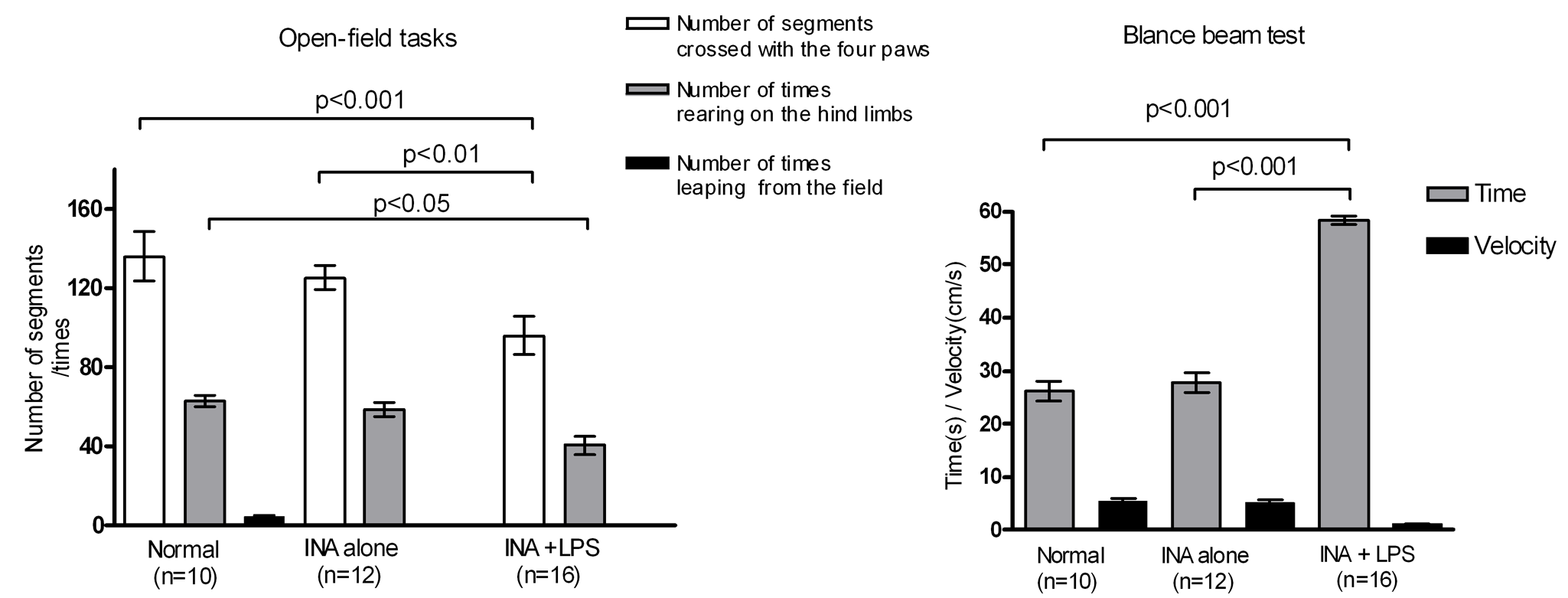

Supplement: Figure S2 — An experimental group (INA immunized, LPS-treated, n = 16) and two control groups (normal, n = 10 and INA immunized alone, n = 12) were studied. Neurological dysfunctions including exploratory behavior and motor coordination, was observed in INA+LPS mice in open field activity and beam walking tasks. (0.20 MB TIF) [file pone.0011124.s003.tif]
